
JOURNAL INFORMATION
==============================
NLM Title Abbreviation: Hist Cienc Saude Manguinhos
Journal ID: Hist Cienc Saude Manguinhos
NLM Journal ID: 9513999
Journal ID: hcsm

História, Ciências, Saúde - Manguinhos

ISSN: 0104-5970
EISSN: 1678-4758
Publisher: Casa de Oswaldo Cruz - Fiocruz

ARTICLE INFORMATION
==============================
PMCID: PMC10481676
PMID: 37672431
DOI: 10.1590/S0104-59702023000100045
Article ID: 01510
Article version: 1
Subjects: Resenhas

Reviewing Flammarion below the Equator

Resenhando Flammarion abaixo da linha do Equador

http://orcid.org/0000-0003-1837-082X Vergara Moema i
i Museu de Astronomia e Ciencias Afins. Rio de Janeiro – RJ – Brasil moema@mast.br
Electronic publication date: 2023 Sep 4
Publication date: 2023
Volume: 30
Electronic Location ID: e2023045
License: This is an Open Access article distributed under the terms of the Creative Commons Attribution License, which permits unrestricted use, distribution, and reproduction in any medium, provided the original work is properly cited.
License URL: https://creativecommons.org/licenses/by/4.0/

Figure count: 1
Table count: 0
Equation count: 0
Reference count: 3

==============================

Quando os editores da revista *História, Ciências, Saúde – Manguinhos* me convidaram para resenhar o livro *Astronomía, literatura y espiritismo: Camille Flammarion en América Latina* (Ramírez, Sevilla, Nieto-Galan, 2022), olhei nas estantes os meus livros do autor. Tinha me passado completamente despercebido que o romance *Urânia* (Flammarion, 1966) foi editado pela Federação Espírita Brasileira. Há muito tempo trabalho com a história da divulgação da ciência no Brasil, em especial a astronomia, e não sabia da relação entre Flammarion e o espiritismo. Para quem estuda a história da divulgação da ciência, o nome de Flammarion é bastante conhecido, inclusive suas incursões pela literatura. Nesse sentido, cabe lembrar que a doutrina espírita pode ser considerada uma derivação da matriz cientificista bem à moda dos Oitocentos. Muitos médicos e cientistas se converteram ao espiritismo, como foi o caso de Camille Flammarion.

O livro também tem o mérito de incluir o Brasil na América Latina, o que pode parecer óbvio do ponto de vista geográfico e cultural, mas grande parte dos brasileiros ainda considera latino-americanos os outros, e não a si próprios.

Nos vários capítulos, observo a influência da ideia de circulação tanto de Robert Darnton, na história da leitura, quanto de James Secord, na história da ciência.

Outra presença constante é Roger Chartier, para quem o processo de tradução é em si um ato criativo e produtor de conhecimento. E como os textos de Flammarion foram traduzidos na América Latina, o papel dos tradutores é explorado em vários capítulos do livro. Gostaria de chamar a atenção ainda para o caso da jornalista colombiana Soledad Acosta de Sampler (1833-1913), que produziu nas últimas décadas do século XIX, uma rara presença feminina no livro trazida por Paola Andrea Benavides. Sampler foi instrumental no debate entre religião e ciência, ao traduzir os trabalhos de Flammarion sobre a pluralidade dos mundos. Para ela não haveria problema de os católicos aderirem às ideias do francês, uma vez que reconhecessem que Deus era o criador daqueles mundos.

Na introdução, os autores afirmam que a divulgação da ciência realizada por Flammarion foi utilizada nos países latino-americanos, na ânsia de sua elite por promover o "progresso" e a "civilização". No livro, esses termos estão sem as aspas; contudo, para uma publicação do século XXI, estes termos não podem mais ser utilizados como se fazia no século XIX ou mesmo no século XX. É necessário pesar o custo social desses processos de "progresso" e modernização, uma vez que frequentemente ocorreram por meio da violência e do apagamento das culturas e vida dos povos originários e de origem africana. E isso é o outro lado da moeda que precisa ser evidenciado pelos cientistas sociais, pois a repetição sem crítica desses conceitos só reforça um eurocentrismo atávico.

O primeiro capítulo, escrito por Agostí Nieto-Galan, teria a função de apresentar Camille Flammarion aos leitores. O autor, entretanto, está tão preocupado em forçar as tintas em relação às ideias da astronomia popular de Flammarion e o espiritismo, que pouco se conhece da biografia do astrônomo francês. Para Nieto-Galan, a astronomia da época era uma ciência que buscava a harmonia entre os mundos celeste e terreno, um escapismo contra os problemas do cotidiano. O que não corresponde à realidade da prática dos observatórios.

Nesse capítulo, por exemplo, está a informação de que Flammarion trabalhou no Bureau de Longitudes. Segundo Galison (2005, p.105-106), aquele era o lugar fundamental da física e da astronomia do final do século XIX, que se dedicava ao problema da unificação da hora e da determinação das longitudes. Esses serviços da astronomia tinham um impacto grande no cotidiano das nações com as questões de fuso horário, cartografia e trabalhos de delimitação de fronteiras. Nieto-Galan reforça uma ideia de astronomia como fuga da realidade, voltada para a observação do céu como uma prática diletante.

Salvo o capítulo sobre a Colômbia de Juan Sebastian Ruiz que lembra a aplicação da astronomia na cartografia e no estabelecimento da hora. Ruiz aponta também para os usos políticos dessa linguagem pela elite (seja ela liberal ou conservadora), ao buscar modernizar a Colômbia no final do século XIX.

Muito interessante também é o texto sobre a Argentina, de Solidad Queirelhac, pois ressalta como a recepção de Flammarion foi feita por meio do humor nas revistas literárias. Para ele, o espiritismo não era uma religião, mas uma ciência dedicada a fenômenos do espírito. O lado "metafísico" do francês ficou restrito às publicações do tema na Argentina. Assim, Flammarion dá um verniz científico ao espiritismo. Nesse capítulo é apresentado também Eduardo Holmberg, que escreveu *Viagem maravilhosa do Sr. Nic-Nac ao planeta Marte*, de 1875. Essa obra foi classificada por Queirelhac como uma literatura fantástica, pois Holmberg conciliava o universo do espiritismo com a astronomia em suas páginas. Cabe destacar que esse é o mesmo ano da publicação de *Dr. Benignus*, no Brasil, por Emilio Augusto Zaluar, também influenciado por Flammarion. No livro de Zaluar, seu personagem Dr. Benignus faz uma viagem ao interior do Brasil e encontra extraterrestres vindos do Sol.

Sobre o Brasil, escreveram Maria Rachel Fróes Fonseca e Kaori Kodama. O texto parte da institucionalização da astronomia no século XIX, mostrando como cientistas e literatos trabalhavam em conjunto no projeto de divulgação da ciência. Assim, escritores como Zaluar estão ao lado dos diretores do Observatório Nacional Luiz Cruls e Liais, todos admiradores de Camille Flammarion. As autoras lembram também do amor do imperador Pedro II pela astronomia como um dos fatores para sua popularização.

Concluída a leitura do livro fica clara a importância da influência de Camille Flammarion na América Latina da segunda metade do século XIX até o começo do XX. As experiências locais variaram principalmente quanto ao espiritismo. No Chile, fica evidente a resistência católica à nova doutrina/religião, em contraste com o Brasil e a Argentina, onde isso ocorria sem muito atrito com a religião hegemônica. Já no caso da inspiração na literatura, em especial na ficção científica, sua presença é incontestável juntamente com Júlio Verne.

Ao ler o livro, vejo que a popularidade da astronomia nos séculos XIX e XX em diversos países da América Latina teve a ajuda do espiritismo e da literatura. Elementos não muito frequentes em análises sobre o tema, mas que contribuíram para que a ciência tivesse peso nos processos em que muitas vezes iam além das questões da produção do conhecimento, como os usos de grupos políticos para se legitimar no poder.

AGRADECIMENTO

Agradeço a Isabela Calil a revisão da resenha.


REFERÊNCIAS

FLAMMARION Camille Urânia Rio de Janeiro Federação Espírita Brasileira 1966
GALISON Peter Os relógios de Einstein e os mapas de Poincaré Lisboa Gradiva 2005
RAMÍREZ Verónica SEVILLA Eliza NIETO-GALAN Agostí Astronomía, literatura y espiritismo: Camille Flammarion en América Latina Santiago RIL Editores; Universidad Adolfo Ibanez 2022
